# Avoiding Pitfalls in Thermal Dose Effect Relationship Studies: A Review and Guide Forward

**DOI:** 10.3390/cancers14194795

**Published:** 2022-09-30

**Authors:** Carolina Carrapiço-Seabra, Sergio Curto, Martine Franckena, Gerard C. Van Rhoon

**Affiliations:** 1 Department of Radiotherapy, Erasmus MC Cancer Institute, University Medical Center Rotterdam, 3015 GD Rotterdam, The Netherlands; 2 Department of Radiation Science and Technology, Faculty of Applied Sciences, Delft University of Technology, 2629 JB Delft, The Netherlands

**Keywords:** hyperthermia, thermal dose effect relationships, hyperthermia heating systems, thermometry, reporting guidelines

## Abstract

**Simple Summary:**

Hyperthermia, heating tumours to 39–44 °C, is a therapy used for cancer treatment. The temperature measured during treatment can be used to obtain the thermal dose delivered to the patient, and this can be correlated with the treatment outcome or toxicity (thermal dose effect relationship). While some clinical studies have found a correlation between thermal dose and treatment outcome or toxicity, others have not. In this review, we assessed the literature to understand possible differences in the hyperthermia treatment characteristics that could have impacted the results of the published studies. We found that fundamental information was not or not uniformly reported in the clinical studies investigating thermal dose relationships. Based on this, we propose definitions and reporting guidelines that have the biggest potential to solve the identified pitfalls. We hope that by standardising the reporting of hyperthermia treatment characteristics, progress in thermal dose effect relationships will be made.

**Abstract:**

The challenge to explain the diffuse and unconclusive message reported by hyperthermia studies investigating the thermal dose parameter is still to be unravelled. In the present review, we investigated a wide range of technical and clinical parameters characterising hyperthermia treatment to better understand and improve the probability of detecting a thermal dose effect relationship in clinical studies. We performed a systematic literature review to obtain hyperthermia clinical studies investigating the associations of temperature and thermal dose parameters with treatment outcome or acute toxicity. Different hyperthermia characteristics were retrieved, and their influence on temperature and thermal dose parameters was assessed. In the literature, we found forty-eight articles investigating thermal dose effect relationships. These comprised a total of 4107 patients with different tumour pathologies. The association between thermal dose and treatment outcome was the investigated endpoint in 90% of the articles, while the correlation between thermal dose and toxicity was investigated in 50% of the articles. Significant associations between temperature-related parameters and treatment outcome were reported in 63% of the studies, while those between temperature-related parameters and toxicity were reported in 15% of the studies. One clear difficulty for advancement is that studies often omitted fundamental information regarding the clinical treatment, and among the different characteristics investigated, thermometry details were seldom and divergently reported. To overcome this, we propose a clear definition of the terms and characteristics that should be reported in clinical hyperthermia treatments. A consistent report of data will allow their use to further continue the quest for thermal dose effect relationships.

## 1. Introduction

Hyperthermia, referring to an increase of the target temperature between 39 and 44 °C, is a potent oncological modality when used in combination with radiation therapy and/or chemotherapy [1,2,3,4,5]. Historically, hyperthermia (HT) has had a mixed profile: after encouraging pre-clinical studies (in vitro and in vivo), clinicians adopted HT with radiotherapy (RT) with various encouraging successes. Several clinical studies, for a wide range of tumour locations, showed that adding HT to RT resulted in an improved treatment outcome [6,7,8]. Despite this evidence, different factors hampered a wider acceptance of HT, of which insufficient control of the heating quality by the available HT systems, non-homogenous treatment delivery, as well as labour-intensive HT treatments are worth mentioning. In addition, the introduction of a thermal dose parameter characterising the clinical response was considered fundamental, not only to deliver HT to a prescribed quantity and measurable quality, but also to anticipate patient benefit. Sapareto and Dewey [9], in 1984, were the first to shed light on the concept of thermal dose. They used the Arrhenius relationship to translate time–temperature data into a cumulative number of equivalent minutes at reference temperature (commonly 43 °C), the CEM43. A modification was, afterwards, included to describe the CEM43 exceeded by x% (e.g., 90%, 50%, and 10%) of the measured temperature locations over a certain period, i.e., CEM43Tx.

Although CEM43 and its variants CEM43Tx account for the dominant biological effect at high temperatures, i.e., cytotoxicity, in most clinical studies, the measured tumour temperatures reported were lower than the intended 43 °C. Since biological mechanisms vary according to the achieved temperatures, at this lower temperature, apart from cytotoxicity, other biological effects, such as perfusion and enhanced oxygenation, are presumably dominant [10,11]. Detailed explanations of the dependence of the biological mechanisms in relation to temperature were reported in [4,5,12]. To address this and other lacunae, more parameters have been proposed over the years. These ranged from basic temperature values (minimum, average, maximum) and index temperatures (tumour temperatures exceeded by x% of the measured temperature points, Tx) to TRISE [12] and the area under the curve (AUC) [13]. Therefore, a wide range of temperature and thermal parameters has been used to report clinical outcome [14]. Despite this variability, all these quantities are, nevertheless, obtained from measured temperatures, emphasising the fundamental role of thermometry in HT treatments.

Thermometry provides crucial information of the temperature history over the course of the HT treatment. This allows improved control of the temperature distribution in the patient by instantaneous adjustments of the various control parameters (e.g., power, phase) of the heating system. Further reasons for registering temperature include the possibility of (i) assessing clinical effectiveness and (ii) preventing toxicity. From the temperature distribution, both the biologic effectiveness and the incidence of thermal toxicity can be estimated. In other words, thermometry is regarded as a dosing and safety control procedure. The great impact of the extent and rigour of applied thermometry in the accuracy of measured temperature fields has been previously described [15,16]. A lack of quality assurance, in particular, insufficient thermometry [17], inadequate sampling of the target volume, and the acquisition rate of temperature measurements [18] were factors causing a wide variation in reported doses. This, consequently, interferes with the identification of thresholds for effectiveness and toxicity, ultimately influencing the determination of a thermal dose parameter [19].

Clinical practice procedures for proper assessment of the temperature distribution have been addressed in different quality assurance guidelines [15,20,21,22,23,24,25]. However, few objective criteria by which to judge the quality of the acquired thermometry data exist. Various challenges are seriously constraining consistent and robust application of thermometry: placement of probes (surrogate intraluminal or superficial locations instead of intratumour positioning), number of measurement points, spatial distribution of measurement points, and the acquisition rate of measured temperatures [26,27]. In addition, the inherent variability in procedures (e.g., between institutes, protocols, tumour sites, heating technology systems), the experience of staff and the extensive technical developments introduced in HT over the years should also be considered. Hence, the quality and quantity of acquired temperature measurements are expected to influence the reliability and representativity of the calculated thermal parameters.

The present study intends to summarise and report clinical and technical characteristics that are expected to influence temperature and thermal dose parameters and, thus, thermal dose effect relationships. Firstly, a systematic review was conducted to select HT clinical studies investigating the associations of temperature and thermal dose parameters with treatment outcome and/or toxicity. From these, different HT-related characteristics were retrieved and analysed. The influence of the accuracy, representativity, and ultimately, reliability of temperature data acquisition on temperatures and thermal dose parameters is discussed. Finally, suggestions for objective and effective HT treatment reports are proposed, which may provide data on (new) temperature and thermal dose parameters able to guide treatment and, ultimately, predict treatment outcome and toxicity.

## 2. Materials and Methods

### 2.1. Search Strategy

The systematic review was conducted in accordance with the Preferred Reporting Items for Systematic Reviews and Meta-analysis (PRISMA) guidelines [28] and registered with the Research Registry (reviewregistry1453). No ethics committee approval was required for the present systematic review.

Four databases, namely EMBASE, Web of Science, Cochrane library, and clinicaltrials.gov, were searched. An initial search was performed in EMBASE according to Bramer et al. [29] to find the best search terms. Synonyms for HT and thermal parameters in those articles were used as entrance terms in the final search. For each database, an individual search was performed, using the same key search sentence (Supplementary Results). The search was limited to English articles, but it was not restricted to date. A filter for full-length published papers and clinical studies was applied. The last search was performed on 23 June 2022.

### 2.2. Inclusion Criteria

Both single-arm and 2-arm studies, randomised and non-randomised, and retrospective and prospective fulfilling the following criteria were included: (1) cancer treatment with HT and RT (these could also include surgery, concurrent chemotherapy, and brachytherapy); (2) associations of temperature or thermal parameters with treatment outcome or toxicity were investigated.

### 2.3. Study Selection

**Figure 1:**
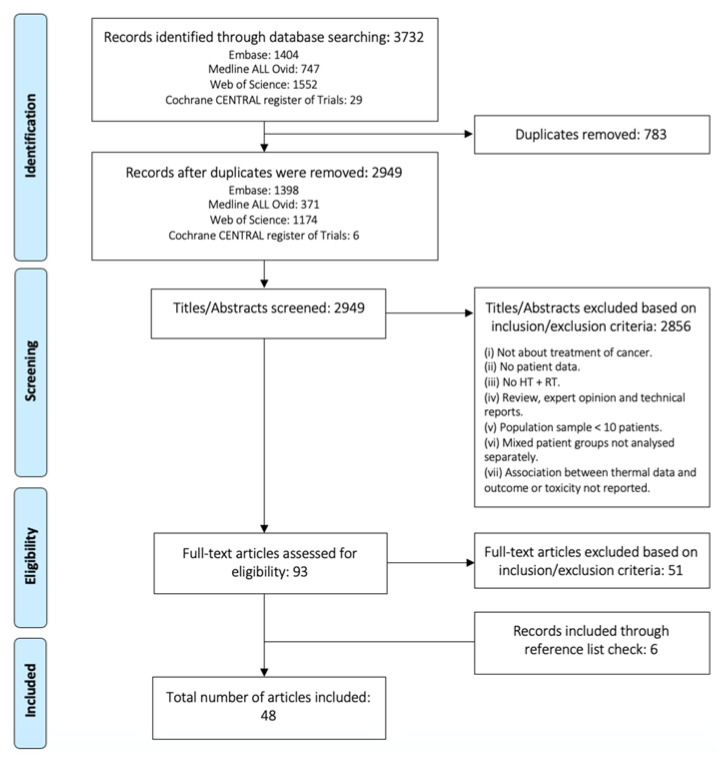
Flow chart indicating the study selection procedure according to PRISMA statement [28].

After exclusion of duplicates, articles were screened according to their titles and abstracts. Review articles, expert opinions, case reports, use of nanotechnology, treatment for non-malignant lesions, and studies with a population sample of less than ten patients were excluded. Articles with mixed patient groups for which the treatment characteristics and endpoints (treatment outcome and toxicity) were not documented separately were excluded. The reference lists of all the included articles were searched for additional literature. Therefore, from the 93 full-text articles considered for detailed study, 51 were excluded. This process is illustrated in Figure 1.

### 2.4. Data Extraction and Evaluation

The primary endpoint of interest was the association of temperature and thermal dose parameters with treatment outcome (e.g., complete response (CR), disease-free survival (DFS), disease-specific survival (DSS), relapse-free survival (RFS), local control (LC), pathological complete response (pCR)) or toxicity. Treatment-related categories were defined, and the corresponding details were retrieved from all selected studies and tabulated. Generic information included the type of cancer, number of patients, RT dose, and the additional use of concomitant chemotherapy. Since a variety of different tumour types were treated within the same study, the categories of superficial and pelvic were also defined. It is important to note that breast lesions could also be included within the superficial tumour category. Hyperthermia treatment parameters comprised total treatment duration, number of sessions per week and per patient, and sequence (i.e., before or after RT and/or chemotherapy). The used HT heating system category was based on the technology reported by the studies.

**Figure 2:**
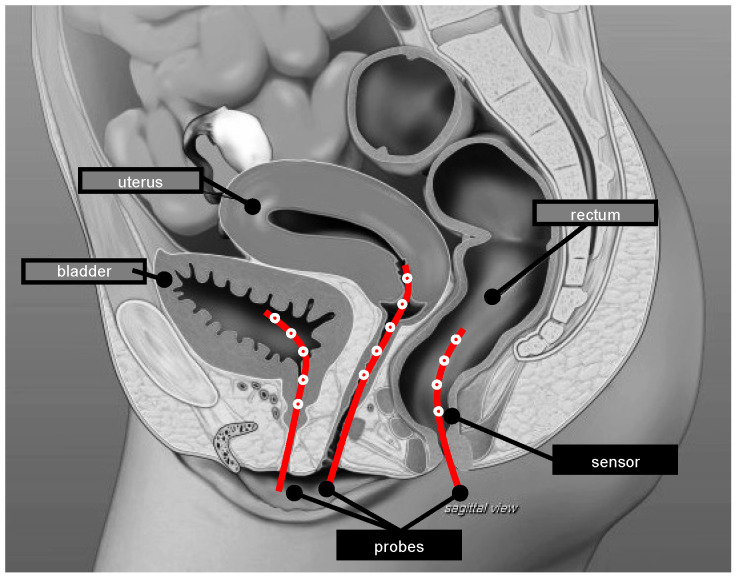
Schematic illustration of the difference between temperature probes and sensors for the pelvic region. The number of probes is three and the number of sensors 14. The number of sensors per probe ranges between four and five.

Considering the technological aspects of performed thermometry, the used temperature measurement technology and the acquisition rate were assessed. When temperature acquisition was reported as non-continuous, this was set to the non-continuous group; when mapping was reported, this was set to the mapping group. For these two groups, the time interval between the sequential measurements, i.e., temperature acquisition rate, when reported was also retrieved. The continuous group included studies reporting continuous acquisitions and acquisitions where the time between measurements was up to one minute. The invasive thermometry placement was also retrieved. The invasive or minimally invasive thermometry category included intratumour, interstitial, and intraluminal measurements. Superficial thermometry was defined as being at the surface. For the analysis of the number of probes and sensors, a probe was considered as a line of measurements and a sensor as the temperature-specific measurement point (Figure 2). Both temperature and thermal dose data and non-significant or significant associations with treatment outcome and/or toxicity were assessed. Given the variability of the values reported for each of the HT characteristics, data were processed in order to homogenise and group the values, when appropriate.

The data of the articles were extracted independently by 1 author (C.C.S), and in case of ambiguity, the shortlisted articles were reviewed by the co-authors to ascertain the correctness of all entries.

### 2.5. Critical Appraisal and Risk of Bias

The risk of bias was assessed using the Quality Prognosis Studies (QUIPS) tool [30]. Six different domains were evaluated: patient selection, study attrition, prognostic factor measurement, outcome measurement, confounding measurement and account, and statistical analysis and reporting. Each domain was scored with a “low,” “moderate,” or “high” risk of bias. The studies were considered to be of high quality when the bias was rated as low or moderate with respect to almost all domains.

## 3. Results

**Figure 3:**
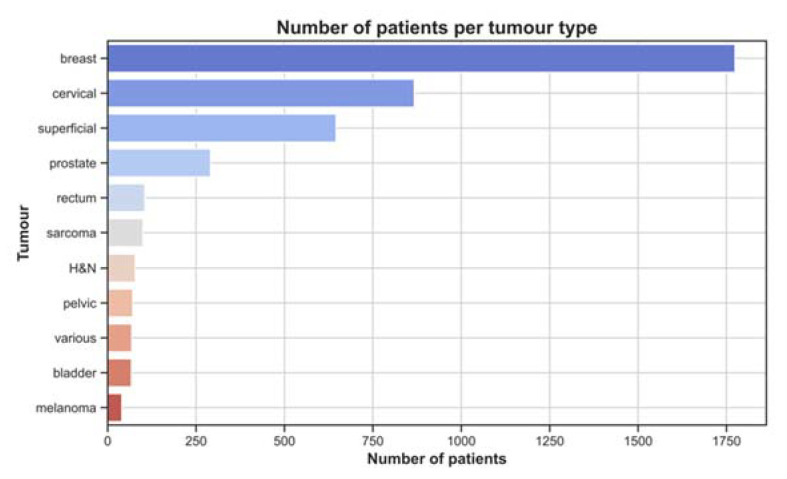
Number of patients included in the review per tumour type.

A total of 2949 articles were identified through four different databases. Forty-eight studies were included in the systematic review (Table 1). These report a total of 4107 patients with a wide range of tumour pathologies. Figure 3 shows the number of patients included in the review per tumour type. This figure shows that patients were mainly treated for breast cancer, followed by cervical cancer and superficially located tumours. All the data retrieved from the included articles are presented in Supplementary Table S3. The study selection and risk of bias assessment using the QUIPS tool can also be found in the Supplementary Results.

**Figure 4:**
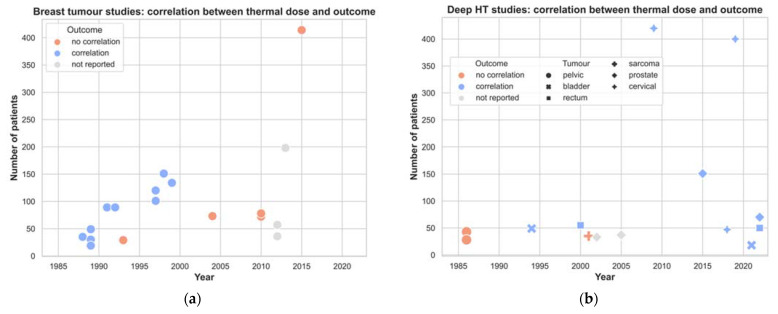

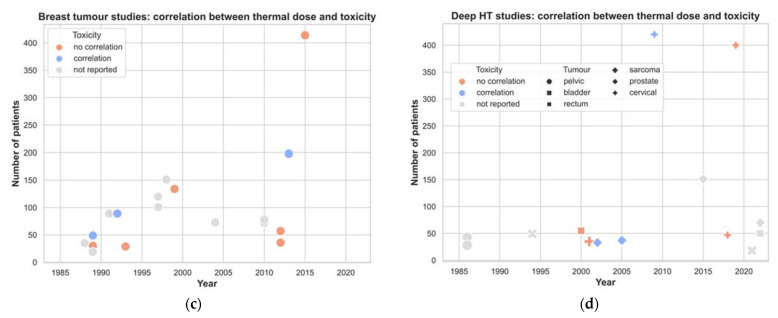
Overview of published studies on thermal dose effect relationships from 1985 to the present separated into superficial HT (breast tumours) and deep HT. Both treatment outcome and toxicity endpoints are shown: (**a**) reported association of thermal dose with outcome for superficial HT in breast tumours and (**b**) for deep HT; (**c**) reported association of thermal dose with toxicity for superficial HT toxicity in breast tumours and (**d**) deep HT.

All included articles analysed the ability of different temperature and thermal dose parameters to predict treatment outcome or toxicity. Treatment outcome was the investigated endpoint in 90% (44/48) of the articles, while toxicity was investigated in 50% (24/48) of the articles. Significant associations between temperature-related parameters and treatment outcome were reported in 63% (30/48) of the studies, while those between temperature-related parameters and toxicity were reported in 15% (7/48) of the studies. The distribution of these associations over the years is shown in Figure 4, for both superficial and deep HT. For deep HT, the data for various tumour pathologies are presented, whereas for superficial HT, the results are only for breast cancer, since these concern most of the studies. It is possible to see that the ability to demonstrate a thermal dose effect relationship varies over time and shows opposite trends for superficial and deep HT.

### 3.1. Treatment Characteristics

The total number of patients per study ranged between 17 and 420 (mean ± sd: 85.6 ± 94.1). The mean RT dose delivered was 43.2 ± 13.8 Gy (range 15–76 Gy), and chemotherapy was combined with RT+HT treatment in 10% of the studies. Superficial HT treatments were reported in 69% (33/48) of the articles, while deep HT treatments in 29% (14/48). One study (2%) reported cohorts treated with both superficial and deep HT. Different HT systems were used: microwave (47%), radiofrequency (16%), capacitive (10%), and ultrasound (6%). Twenty percent of the studies reported HT applied with more than one system (Table 2). This was because of wide range of tumour sizes and depths were treated within the same patient cohort and the technological developments implemented throughout the study.

In 90% of the cases, treatments were given in a scheme of one or two sessions per week, each taking on average 56.6 ± 15.8 min (range: 30–90 min). Superficial treatments lasted 53.7 ± 11.7 min, and deep treatments lasted 62.8 ± 22.0 min (mean ± sd). The mean number of HT sessions per patient was five (range: 2–10 sessions). Hyperthermia was mostly delivered after RT and/or chemotherapy (69%), with a time interval of up to 60 min (76%) between therapies. In seven (15%) studies, HT was before RT/CT, and in five (11%), the order was not reported. The other category includes two studies in which RT+HT was delivered simultaneously, while one study delivered HT both before and after RT. This information is summarised in Table 2.

### 3.2. HT System and Thermometry

**Figure 5:**
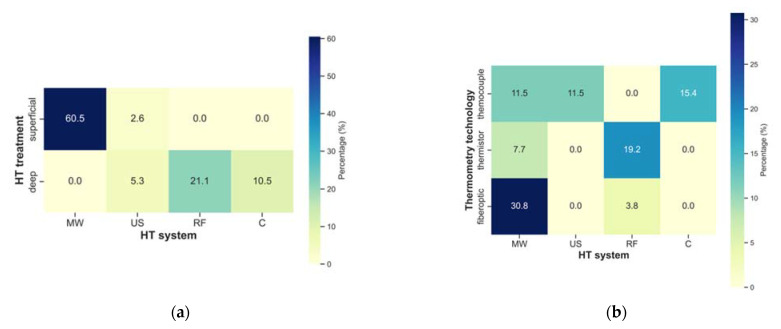

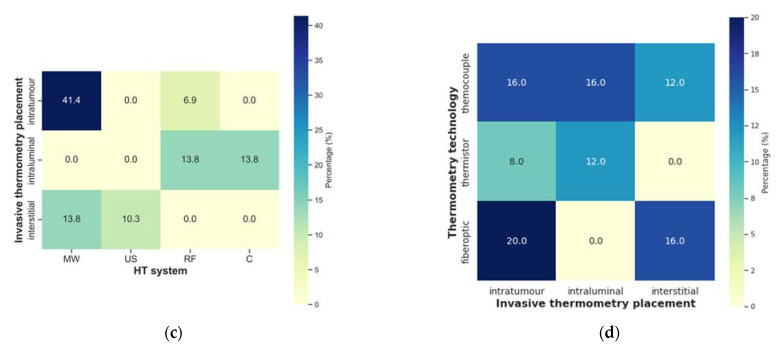
Heatmaps showing the relative frequency of the combination of (**a**) HT treatment vs. HT system, (**b**) thermometry technology vs. HT system, (**c**) invasive thermometry placement vs. HT system, and (**d**) thermometry technology vs. invasive thermometry placement. (MW: microwave, US: ultrasound, RF: radiofrequency and C: capacitive).

The frequency assessment of different HT characteristics and thermometry is provided in Figure 5. Observing the HT treatment type, i.e., superficial or deep against the HT system, microwave was the most-used system for superficial treatments. Conversely, radiofrequency was the mostly adopted system for deep treatments. Regarding the HT system and thermometry technology, microwave was mainly combined with fibreoptic probes. This might be due to the potential interaction of the metal parts of the thermocouples with the electric fields. Additionally, the radiofrequency (RF) interference of the electronic readout of the temperature signal also makes temperature measurements with thermocouples during high-frequency treatments challenging. In contrast, the RF-immune “Bowman” thermistors [75] were more used with RF devices. With microwave systems, probes were placed intratumourally, while for RF and capacitive systems, probes were placed intraluminally. Ultrasound systems were always combined with interstitially placed probes. Thermocouples seem to be applied in all invasive placement settings, while fibreoptic probes are used for intratumour and interstitial measurements and thermistor probes in intratumor and interstitial measurements.

Invasive or minimally invasive thermometry was performed in all studies and superficial thermometry in 64%. Articles not reporting this information were assumed to not have performed it. The use of a combination of different thermometry technologies was reported in 30% of the studies, while thermocouple, fibreoptic, and thermistor technology was applied in 23%, 19%, and 13%, respectively. The placement of (minimally) invasive thermometry was in 52% of studies intratumourally and in 19% interstitially and intraluminally. Concerning the acquisition, 27% of studies performed non-continuous temperature acquisition (including mapping). Continuous acquisition was performed in 23% of the studies. Combined acquisitions (e.g., continuous in specific probes and mapping in others) was performed in 23% of the studies. Twenty-seven percent of the studies omitted this information. Regarding the rate, 23% of the articles reported temperature acquisition in intervals between one and five minutes, while only 13% acquired temperature data in intervals of more than five minutes. A concerning fact is that information on the rate of temperature acquisition was missing in 41% of the studies.

**Figure 6:**
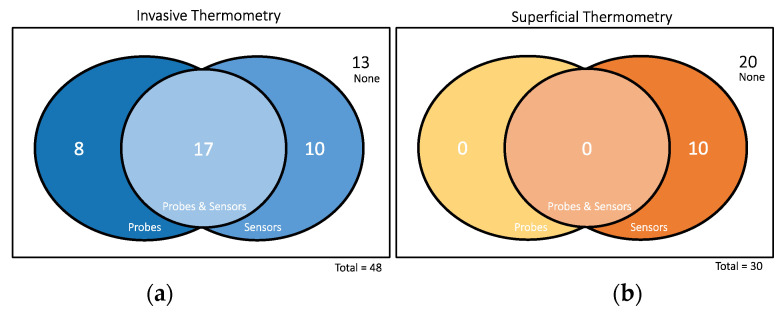
Number of articles reporting the number of probes and sensors for (**a**) invasive thermometry and (**b**) superficial thermometry.

When reported, the mean number and standard deviation of invasive probes and sensors was 2.3 ± 1.0 and 9.5 ± 7.2, respectively. For superficial thermometry, only values of sensors were reported, being the mean and standard deviation values 10.9 ± 12.3 (Table 2). It should be pointed out that the mentioned values were calculated from a relatively small number of instances, as can be observed in Figure 6. Only 17 out of the 48 studies reported both the number of probes and sensors used for (minimal) invasive thermometry, while none provided this information in the total of 30 studies performing superficial thermometry. The report of the number of sensors is more frequent in invasive thermometry. In superficial thermometry, the number of sensors was reported in only ten studies, out of 30 that performed superficial measurements.

### 3.3. Temperature and Thermal Dose Parameters

**Figure 7:**
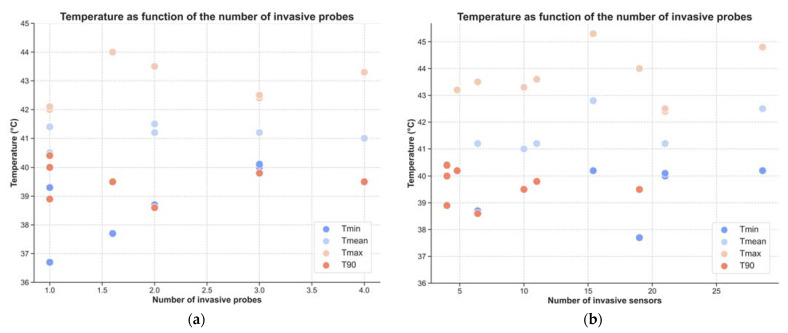
Temperature parameters as a function of the number of invasive probes (**a**) and invasive sensors (**b**).

Data from studies reporting both the number of invasive probes or sensors and the mean or median temperatures were used to plot temperature against the number of invasive probes (Figure 7a) and the number of invasive sensors (Figure 7b). From the first figure, trends are difficult to perceive, specifically for maximum temperature (Tmax), mean temperature (Tmean), and T90. Minimum temperatures (Tmin) seem to increase with the number of probes. The same was found for all temperatures when the number of sensors was analysed: higher temperatures appear to be linked to a higher number of sensors. It is noteworthy that the total number of reports retrieved from the literature is particularly limited, i.e., maximum of seven reports for each parameter. Moreover, the temperatures reported were obtained not only from tumour locations, but also from all locations where measurements were performed.

**Figure 8:**
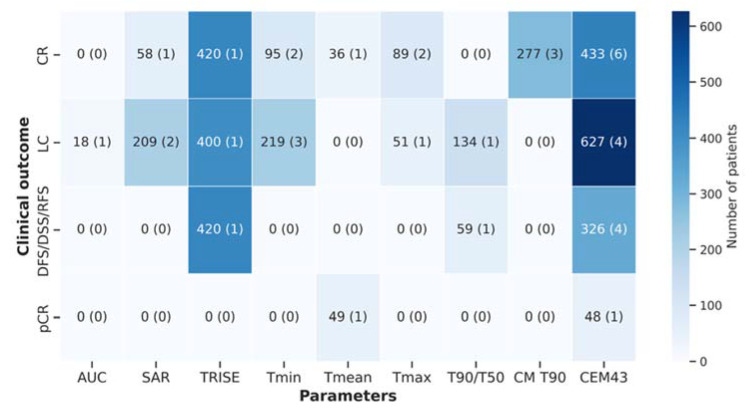
Heatmap showing the association of clinical outcome with temperature and thermal dose parameters in terms of the number of patients. Brackets correspond to the number of studies. CR: complete response; DFS: disease-free survival; DSS: disease-specific survival; RFS: relapse-free survival; LC: local control; pCR: pathologic complete response; AUC: area under the curve; SAR: specific absorption rate; Tmin: minimum temperature; Tmax: maximum temperature; T90/T50: temperatures exceeded by 90%/50% of the measured temperature points; CM T90 cumulative minutes T90; CEM43: cumulative minutes at 43 °C.

Trends towards the association of temperature and thermal parameters with different clinical outcomes are shown in Figure 8. CEM43 was the parameter mostly investigated as the treatment outcome predictor, followed by Tmin. While the former was mainly correlated with CR, LC, and DFS/DSS/RFS, the latter was only associated with CR and LC (Figure 8). Conversely, when evaluating for sample size, i.e., the number of patients, CEM43 was mostly associated with LC. Although in the majority of studies, CR was the clinical endpoint used, LC was the endpoint that comprised more patients in total. DFS/DSS/RFS and pCR are less predicted by the different temperature and thermal dose parameters. Concerning pCR, this parameter was not extensively investigated, since only a few studies included surgery to remove the tumours after RT+HT, and thus, only those can evaluate the specimen for histopathology.

## 4. Discussion

Various thermal dose parameters have been reported in the literature over the years. Our study provides an extensive quantitative elaboration of the published literature to identify the most relevant temperature and thermal dose parameters to predict treatment effectiveness. Our analyses cover all the studies reporting associations of different temperature and thermal dose parameters with two specific endpoints, treatment outcome and toxicity. As far as we know, this is the first study to investigate the predictive impact of various clinical and technical factors on thermal dose relationships [14,27]. The identification of these factors could shed light on a possible framework for setting up prognostic data collection studies, which will ultimately improve our understanding of thermal dose effect relationships. In the discussion, we try to establish the relative contribution of each clinical and technical factor and to provide a guide on how to proceed. Complementary to our study, in a recent review on the clinical evidence to guide HT treatments based on the measured temperature and thermal dose parameters, Ademaj et al. [76] concluded that the limited international standards for the delivery of HT in the clinical setting result in a large variability in the reported thermometric data. As a consequence, developing guidelines for the delivery of an adequate thermally dosed HT treatment is fundamental.

### 4.1. Impact of Time

Studies that investigated the quality of the applied HT treatment by reporting the measured temperature distribution or the delivered thermal dose have been initiated from the early clinical application of HT and span a period of more than 30 years. Inevitably, the standard practice of cancer treatment has evolved over such a long period. Besides, the patient treated currently with HT is also different from the patient treated in the 1990s. The impact of evolving cancer treatment is, to some extent, also reflected in the reported thermal dose effect studies published in the literature over time.

It is interesting that the ability to demonstrate a thermal dose effect relationship varies over time and shows opposing trends for superficial and deep HT. As shown in Figure 4a, the ability to demonstrate a thermal dose effect relationship for treatment outcome in breast cancer has reduced over the last two decades. An explanation for this finding is not straightforward. A feasible explanation could be that current patients with recurrent breast cancer present with a different tumour load. Moreover, the treatment of breast cancer recurrences with RT+HT became the standard of care in many countries (e.g., the Netherlands, Germany) around the year 2000 following the publication of Vernon et al. [77]. This led to less studies investigating its effectiveness and, therefore, thermal dose effect relationships. It is interesting to note that, for the fifteen-year period between 1985 and 2000, only five studies investigated the toxicity, while for the five-year period between 2010 and 2015, four did. This shows a shift in the community’s interest, since more recently, the focus is avoiding toxicity in breast cancer treatments, due to it now being considered a standard treatment.

One can presume that the increased probability over time to demonstrate a thermal dose effect relationship in deep HT (Figure 4b) is likely to be caused by the improvement in RF-heating technology [78,79], as well as its consequent wider usage and wider use of hyperthermia treatment planning. Until 1990, deep HT was mostly applied by capacitive systems or early radiative annular phased arrays, which did not provide the ability to steer the energy into the tumour. Nevertheless, after the positive results of the randomised control phase III trial, i.e., Dutch deep hyperthermia study [80], the interest in deep HT when added to RT grew. This led to an increase in the number of clinically available systems. The experience built-up over the following years and the scientific interest to find the minimal thermal dose required to heat deeply located tumours might have stimulated the publication of studies in thermal dose effect relationships for deep HT. As they were also performed within a single institute, the HT treatments were applied according to the same protocol, i.e., homogeneous heating strategy and temperature monitoring. A similar observation cannot be made with regard to finding a thermal dose effect relationship with toxicity (Figure 4d). The ability to find such a relationship is, historically, low. The latter is not surprising, as in deep HT, temperature registration in normal tissue at areas at risk for toxicity is not considered standard practice.

### 4.2. Treatment Characteristics

Radiotherapy dose influences treatment outcome for different pathologies, not only when applied alone, but also when applied in combination with HT [52,59,64]. In this investigation, the obtained radiation dose varied over a large range (15–76 Gy). This is explained by the fact that different types of tumours were included, which were inherently treated with different total doses, as well as fractionations, schedules, and intents (radical vs. palliative). Overall, the fundamental information on the characteristics such as the fraction size, overall time (e.g., weeks), boost, and effective radiation dose was not widely reported. Hence, the contribution of RT dose as an independent variable in the thermal dose effect relationship studies could not be established. Concerning chemotherapy, it was seen that four out of a total of five studies that added this therapy to RT+HT were able to find a thermal dose effect relationship. However, since only five studies (10%) included chemotherapy, this evidence should be further investigated.

The number of HT treatments has been extensively investigated to better understand the thermotolerance phenomenon [38,49,81]. Thermotolerance, i.e., a temporary heat resistance following a prior heat treatment, is induced during an HT session, resulting in a transitory resistance to temperature increase in subsequent HT sessions, thereby influencing the thermal sensitivity of tissues to subsequent heat treatments. Clinically, HT is delivered once or twice a week to avoid ineffective HT sessions due to thermotolerance. In view of the complex interplay of the thermoradiobiological interactions, a standardised treatment protocol applied for a single tumour pathology in a clinical thermal dose effect study is desirable. This results in more homogeneous thermal enhancement and, thus, allows better comparison and, eventually, more insights into such complex process.

Treatment duration is mainly associated with the type of HT, i.e., superficial HT treatments or deep HT treatments. Currently, the former is usually delivered in 60 min, while the latter lasts approximately 90 min. However, when separating the total HT duration per type of treatment (superficial versus deep), this was not evident. The explanation comes from the fact that, in the early years, many treatments had a shorter duration of 30–45 min for superficial [32,35,39,40,41,42,43,44,61] and approximately 45–60 min for deep HT [17,34,51,58,60,62,70,71,73]. Prostate cancer is usually treated with a shorter duration than bladder and cervical cancers. Moreover, tumours treated with capacitive systems are also heated for shorter periods of time than those heated with RF systems. Despite the variability expected on the duration between superficial and deep treatments, the number of HT sessions per patient seems to be the same for all studies (approximately five sessions). This is also seen for the sequence of treatments, which mainly consists of RT followed by HT.

### 4.3. Impact of HT Systems

HT equipment should be tailored to the patient’s requirements, including tumour size, shape, and depth. However, early clinical studies were part of the discovery journey, and most first-generation HT devices were not capable of achieving such requirements, compared to a much better matching to these requirements with improved later-generation HT devices [18,79,82]. The first generation of microwave applicators, for instance, could only achieve depths of ≤4 cm below the skin and could only effectively heat small lesions, since only 30–60% of the aperture face could be heated [83,84,85]. For deep-seated tumours, patient had to be shifted within the applicator to change the focus location when electronic steering of the focus could not be performed. In these devices, overheating outside of the target volume (hotspots) was also a significant and treatment-limiting factor [86]. With the goal to increase control over power deposition at depth, the latest generation of radiative deep heating devices has a higher number of antennas, which results in a large number of degrees of freedom, i.e., the amplitudes and phases of the individual antennas can be controlled to increase the ability to adapt the energy distribution to the target tumour.

Clinical studies demonstrating the dependence of clinical outcome on the HT system used are rare. An exception is the study by van der Zee et al. [57]. They investigated two different heating techniques (433 MHz HT versus 2450 MHz). Despite the fact that the median HT dose parameters were the same, they found that the 433 MHz HT technique resulted in a higher CR rate than 2450 MHz, as well as that the acute and late toxicity was higher for the 2450 MHz technique. The improvement in treatment outcome was clear in tumours larger than 3 cm in diameter, since the lower the frequency, the higher the penetration depth and the larger the heated volume are. Their statistical analyses showed only two parameters to be associated with local control, i.e., tumour size and HT system. The authors reported that the use of a higher number of intratumour temperature measurements with the 433 MHz technique compared with the 2450 MHz technique masked the ability to associate the improved outcome with the higher monitored thermal dose parameters calculated. Hence, their finding was only based on the observation of lesions rather than on temperature or thermal dose parameters. Nevertheless, the study demonstrated that it is quite reasonable to assume that the performance of various HT devices is different, but that the identification of different thermal doses requires an adequately designed temperature measurement protocol.

From our study, we saw that currently available thermal dose effect relationships have mainly been obtained with microwave and RF heating systems. By using constructive interference, this technology provides the ability to heat at deep locations, but the low frequencies translate into relatively large (10–15 cm) diameters of the energy focus. Hence, although the technology provides the possibility to adapt the energy distribution, it does not possess the ability to adapt the temperature distribution at the cm scale. Conversely, for capacitive heating, energy steering is confined to control the total energy output [86]. Although the size of the capacitive plates can also be modified, this must be performed prior to the treatment, not allowing dynamic adaptative control. Hence, the temperature distribution mainly results from the tissue anatomy and perfusion. Regarding ultrasound heating, only three studies applied this technology (without combination with other heating technology), and these were mainly focused on toxicity analysis [60,62,67]. Although this technology is capable of energy control at the sub-centimetre level, more studies should be performed to fully confirm this. Moreover, since ultrasound is capable of investigating the impact of homogeneous versus heterogeneous heating at the tumour level, studies investigating this factor should be extremely valuable.

It should also be borne in mind that the use of a cooling system (e.g., bolus) was not assessed, due to the lack of sufficient information regarding its use, shape, size, and temperature. This has, however, been reported to influence the heating performance of the HT system, specifically for superficial treatments [87].

### 4.4. Quality of Thermometry

Temperature monitoring, ideally continuously, using invasive and/or superficial probes near the tumour and in healthy tissues is used to monitor the quality of the HT treatment. In all of the included studies, thermometry was always performed, yet system and placement characteristics were reported in relatively low detail. A detailed reporting is particularly essential because (1) the commercially available probes differ in terms of the number of sensors and (2) the HT system also influences this. While thermocouples are available from single sensors up to seven to ten sensors, fibreoptics can have up to eight sensors per probe. However, these invasive systems (fibreoptic, thermistor, and thermocouples) only monitor temperature within the volume around the sensors. During the last few years, significant developments have occurred in the field of non-invasive thermometry. Techniques such as X-ray computed tomography, microwave tomography, echo sonography, and magnetic resonance thermometry have been investigated [88]. These methods have the potential to identify hotspot formation in the full region of treatment and elucidate a thermal dose response not only in the location of the probe, but in the full treated region. Currently, magnetic resonance thermometry is the most clinically used non-invasive method. New techniques are being proposed to improve the accuracy and precision of the measurements, which are influenced by tissue motion [88,89].

Concerning the thermometry system, thermocouples have been mainly used, likely because of their low cost, small size, and robustness. Nevertheless, the well-known electromagnetic interference and self-heating of the probes, by induced current and local tissue heating, could still influence the temperature measurements [84,85,86]. Combining suitable RF filters, i.e., absorbing ferrite beads around the probe, with a double-pulsed power technique enables, in most situations, correct reading of the temperature. As reported by Kok et al. [90,91], good thermal contact is essential for reliable temperature readings. In the situation of a poor contact between the temperature probe, catheter, and tissue, a low change of the RF-disturbed temperature readings still exists as the temperature readout is characterised by a relatively long response time due to electromagnetic interference [92]. Minimal interaction with electromagnetic fields is usually expected from fibreoptic and high-resistance Bowman thermistor probes [75,93,94].

The intention of the selected number of thermometry probes and sensors has always been to allow adequate treatment guidance to the target volume and respond to potential hotspots. As a result, representative locations are chosen mainly across the target volume. However, the required number of temperature probes, as well as their location might be different, when the objective is, instead, to gather temperature information to quantify the thermal dose delivered to the treatment field, i.e., both tumour and normal tissue. De Bruijne et al. [27] showed that thermal dose (CEM43T90) is negatively correlated with the number of interstitial points, as well as with tumour maximum diameter. Moreover, the recent study from Bakker et al. [95] showed that the required number of sensors for adequate detection of the maximum temperature to prevent skin toxicity is ≥50 stationary sensors per 400 cm^2^ applicator, i.e., one sensor per 8 cm^2^, a value not reached in any of the studies included in this review. Bakker et al. also indicated that, as an alternative to the ≥ 50 stationary sensors, the use of continuous thermal mapping with approximately 13 sensors could provide similar results. Bearing this in mind, the number of probes and/or sensors used and if they are located in the tumour or healthy tissue should be clearly and always reported. Although fundamental, this information was generally omitted. Ideally, a measure of the target volume sampling should be reported, since this is the only value that provides a measure of sampling adequacy, since an estimate of the number of measurement points in relation to the target volume can be obtained. This would provide a general value that could be used to better compare temperatures acquired through different techniques.

In the study from Arcangeli et al. [32], tumour temperature appeared to be a strong predictor of response, yet only one temperature sensor was used. A discouraging thermal dose was reported by Sapozink et al. [17,34], but the lack of information regarding the number of probes and/or sensors used makes it challenging to form any interpretation about potential issues at all. Identical limitations were present in the studies of Luk et al. [31], Dragovic et al. [37], Li et al. [61], Jones et al. [63], and Oldenborg et al. [65,69], whose studies did not provide any insight into the number of probes and sensors used. Therefore, it is particularly difficult to investigate why some of these studies successfully found thermal dose effect relationships and others did not. On the contrary, Engin et al. [48,49] extensively described thermometry methods performed for invasive temperature monitoring, including not only the thermometry system, but also the mean number of probes and sensors. Further, the range of sensors per probe was also described, which ultimately provides information on the representability of the measured temperatures, i.e., set of linear temperature data points or data points from different probe locations. Catheters were fixed in place during the full course of the treatment, which is an advantage in terms of placement reproducibility for temperature acquisition over the different sessions. Moreover, the study from Juang et al. [96] also exceeded in the report of thermometry, distinguishing between probes with stationary sensors and mapping probes.

As essential as reporting the number of probes and sensors is providing the rate of temperature acquisition, yet this information was generally missing. The temperature acquisition rate is expected to impact the calculation of thermal parameters, since the tissue temperature depends on the local energy removal by blood perfusion and blood perfusion and is tumour- and temperature-dependent. Moreover, the extent and the dynamics of alterations in the tumour blood have been found to depend on the heating speed and homogeneity of the achieved intratumoral temperature [81,97,98]. Therefore, monitoring the temperature with a great number of probes and sensors, during only one or two treatments from a total of five, might not provide the most reliable thermal dose estimation. Yet, Yahara et al. [70] was able to significantly correlate CEM43T90 with DFS for prostate cancer. Moreover, even mapping temperatures two times during a 60 min treatment might also be insufficient to calculate a reliable thermal dose, given the physiological and molecular effects occurring in the tumour. The first published articles performed manual and automatic mapping within the same study, and while the latter would be mostly continuous, the former was performed only once or twice during the treatment [43,44,54].

To better realise the importance of the rate of temperature acquisition on the derivation of thermal parameters, it is essential to provide sufficient information on the temperature acquisition procedure, specifically when mapping is used. If thermal mapping is performed, a single temperature probe can translate into a high number of sensors, but with the consequence that the acquisition rate is low. Each affects the quality of thermometry in a different way. One should also recognise that other unknown factors such as tumour depth, vascularity, and perfusion; adjacent normal structures with variable dielectric properties and blood flow; and the accuracy in patient positioning, as well as individual patient tolerance also play a role in treatment outcome. Thus, achieving a uniform tumour temperature as prescribed may not be always feasible in clinical situations. Heterogeneity in the temperature distribution within the tumour could also be better understood with more and better thermometry, whose information should ultimately be clearly provided when reporting outcomes.

### 4.5. Other Factors

A variable that must be considered is tumour type. In some studies [31,33], different types of tumours were treated using different settings, yet thermal relationships were not analysed separately. Instead, different tumour types should ideally be handled separately [64] and thermal doses reported separately as well. In this sense, Bakker et al. [99] showed that, specifically for breast cancer, temperature and thermal dose during HT had a significant influence on treatment outcome (e.g., CR and LC).

It is essential also to ensure optimal patient-specific applicator settings that yield not only therapeutic temperatures in the tumour, but also minimal normal tissue heating, which is preponderant for the treatment quality. However, in clinical practice, this ideal scenario is hampered by different factors, e.g., patient positioning accuracy and condition. Inaccuracies in patient positioning may lead to deviations from the optimal treatment planning, influencing, thus, temperature measurements and the consequently obtained thermal doses.

CR is the clinical outcome more associated with temperature and thermal descriptors. CEM43 was the parameter significantly associated with CR in a higher number of articles. Moreover, CEM43 was also successfully associated with other clinical endpoints as LC and DFS, the latter being one of the keys in the realm of cancer treatment. This shows the potential of such thermal dose parameter to better predict outcome, if more standardised treatments and thermometry are applied and reported properly.

### 4.6. How to Proceed

The literature clearly shows that a thermal dose exists, but not all studies can reproduce it, because it depends on a wide range of parameters. With the full awareness that the presented results do not unequivocally identify parameters influencing temperature and thermal dose parameters, a clear report, including as many HT characteristics as possible, seems to be required. With greater emphasis on thermometry, we propose in Table 3, a systematic, yet straightforward manner to report the information that is thought to be more relevant.

Firstly, it is fundamental to stress the need for studies on organ-by-organ and disease-by-disease bases. This should be extended to the HT system and thermometry system used, meaning that thermal dose relationships should be investigated in cohorts of patients treated with the same systems, in order to avoid possible differences in the quality of tumour heating. It is interesting to consider the approach of Myerson et al. [41], who verified the appropriateness of the applicator to a patient’s tumour, using specific absorption rate (SAR) distributions. The SAR is a measure of the mass-normalized rate of energy absorption by a biological body [100]. The SAR can be obtained using numerical modelling (finite element models or finite difference methods), based on the type of treatment energy modality and the applicator and patient characteristics or derived from the temperature increase in a specific time [101]. Instead of only serving as a quality assurance parameter to select the best applicator, Myerson also found that patients with tumour SAR coverage ≥ 25% and a minimum CEM43 ≥ 30 yielded an improved tumour control rate. Thus, such a metric might have an added value to thermal dose relationships, and when treatment planning is performed, this should also start to be reported. Moreover, the coupling method should also be described and the temperature at which this was used during treatment.

Thermometry procedures require essential consideration. The performed thermometry should be categorised as invasive or superficial. For each, the used system characteristics and manufacturer should be clearly stated. Within invasive thermometry, the placement of the probes should also be reported. Temperature acquisition can be continuous or non-continuous, while mapping techniques usually fall into the latter. This information should also be unequivocally reported for invasive and superficial acquisitions and complemented by the rate of temperature measurements. In such a manner, equivocal descriptions [13,43,44,55] might be avoided.

A clear definition of the number of probes and sensors should also be described and reported. It is advised to always use the same terminology and avoid synonyms, such as positions, points, maps, and measurements. Thus, we encourage the HT community to use the definition of probes and sensors presented here and to report them accordingly. A measure of the sampling rate, i.e., how many sensors per area of the target should be reported, since this provides a measurement of the adequacy of the target coverage. Temperatures and thermal dose descriptors should be calculated and described separately for invasive and superficial thermometry. A thermal dose should be related to the treatment endpoint if described within the tumour field. Conversely, for superficial monitored positions, thermal parameters should be related to toxicity. Finally, endpoints such as overall survival and DFS should start to be more investigated, as these are the most important.

If reported in a standardised manner, the more information is available, the easier it will be to continue the quest for a thermal dose. As shown by Kroesen et al. [70], thermal dose effect relationships are real, and it should be our task, as a community, to further understand why not all studies can achieve the same conclusion.

## 5. Conclusions

The question of which parameters influence the detection of a thermal dose effect relationship is not easy to answer. Since the clinical studies of the 1980s, the technology for HT has come a long way: great improvements have been attained in both heating and temperature-monitoring systems. This was evident in the present study, by the wide variation within the investigated characteristics extracted from the literature. Evident shortcomings in the reporting of the published studies were also found, which seem to prevent our quest towards the fundamental question of the thermal dose parameter.

It is the authors’ opinion that thermal dose effect relationships exist, and only if studies (1) follow standardised clinical protocols, as well as quality assurance procedures and (2) report results in a complete and uniform manner, this will allow us to further investigate trends on thermal dose effect relationships. A standard definition of probes and sensors was presented, as well as a template to describe different treatment characteristics. Objective and uniform reporting is the basis for evidence-based practice, which ultimately, might be translated into a treatment of high quality.

## Figures and Tables

**Table 1:** Author, year, and title of the articles included in the systematic review.

Author, Year	Title
Luk, 1981 [31]	Clinical experiences with local microwave hyperthermia
Arcangeli, 1985 [32]	Tumour response to heat and radiation: prognostic variables in the treatment of neck node metastases from head and neck cancer
van der Zee, 1985 [33]	Clinically derived dose effect relationship for hyperthermia given in combination with low dose radiotherapy
Sapozink, 1986 [34]	Regional hyperthermia for clinically advanced deep-seated pelvic malignancy
Sapozink, 1986 [17]	Abdominal regional hyperthermia with an annular phased array
Arcangeli, 1987 [35]	Radiotherapy and hyperthermia. Analysis of clinical results and identification of prognostic variables
Gonzalez, 1988 [36]	Chestwall recurrences of breast cancer: Results of combined treatment with radiation and hyperthermia
Dragovic, 1989 [37]	Local superficial hyperthermia in combination with low-dose radiation therapy for palliation of locally recurrent breast carcinoma
Leopold, 1989 [38]	Preoperative hyperthermia and radiation for soft tissue sarcomas: advantage of two vs. one hyperthermia treatments per week
Sannazzari, 1989 [39]	Results of hyperthermia, alone or combined with irradiation, in chest wall recurrences of breast cancer
Seegenschmiedt, 1989 [40]	Superficial chest wall recurrences of breast cancer: Prognostic treatment factors for combined radiation therapy and hyperthermia
Myerson, 1990 [41]	Tumor control in long-term survivors following superficial hyperthermia
Kapp, 1990 [42]	Two or six hyperthermia treatments as an adjunct to radiation therapy yield similar tumor responses: results of a randomized trial
Kapp, 1991 [43]	Hyperthermia and radiation therapy of local-regional recurrent breast cancer: Prognostic factors for response and local control of diffuse or nodular tumors
Kapp, 1992 [44]	Thermoradiotherapy for residual microscopic cancer: elective or post-excisional hyperthermia and radiation therapy in the management of local-regional recurrent breast cancer
Leopold, 1992 [45]	Relationships among tumor temperature, treatment time, and histopathological outcome using preoperative hyperthermia with radiation in soft tissue sarcomas
Leopold, 1993 [46]	Cumulative minutes with T90 greater than tempindex is predictive of response of superficial malignancies to hyperthermia and radiation
Bornstein, 1993 [47]	Local hyperthermia, radiation therapy, and chemotherapy in patients with local-regional recurrence of breast carcinoma
Engin, 1993 [48]	Thermoradiation therapy for superficial malignant tumors
Engin, 1993 [49]	Randomized trial of one versus two adjuvant hyperthermia treatments per week in patients with superficial tumors
Engin, 1993 [50]	Hyperthermia and radiation in advanced malignant melanoma
Masunaga, 1994 [51]	Phase I/II trial of preoperative thermoradiotherapy in the treatment of urinary bladder cancer
Hand, 1997 [52]	Analysis of thermal parameters obtained during Phase III trials of hyperthermia as an adjunct to radiotherapy in the treatment of breast carcinoma
Sherar, 1997 [53]	Relationship between thermal dose and outcome in thermoradiotherapy treatments for superficial recurrences of breast cancer: Data from a phase III trial
Lee, 1998 [54]	Superficial hyperthermia and irradiation for recurrent breast carcinoma of the chest wall: Prognostic factors in 196 tumors
Sneed, 1998 [55]	Survival benefit of hyperthermia in a prospec- tive randomized trial of brachytherapy boost hyperthermia for glioblastoma multiforme
Myerson, 1999 [56]	Simultaneous superficial hyperthermia and external radiotherapy: Report of thermal dosimetry and tolerance to treatment
van der Zee, 1999 [57]	Reirradiation combined with hyperthermia in recurrent breast cancer results in a worthwhile local palliation
Rau, 2000 [58]	Preoperative radiochemotherapy in locally advanced or recurrent rectal cancer: Regional radiofrequency hyperthermia correlates with clinical parameters
Maguire, 2001 [59]	A phase II trial testing the thermal dose parameter CEM43T90 as a predictor of response in soft tissue sarcomas treated with pre-operative thermoradiotherapy
Hurwitz, 2002 [60]	Association of rectal toxicity with thermal dose parameters in treatment of locally advanced prostate cancer with radiation and hyperthermia
Li, 2004 [61]	Local hyperthermia combined with external irradiation for regional recurrent breast carcinoma
Hurwitz, 2005 [62]	Hyperthermia combined with radiation in treatment of locally advanced prostate cancer is associated with a favourable toxicity profile
Jones, 2005 [63]	Randomized trial of hyperthermia and radiation for superficial tumors
Franckena, 2009 [12]	Hyperthermia dose-effect relationship in 420 patients with cervical cancer treated with combined radiotherapy and hyperthermia
Gabriele, 2009 [64]	Radio hyperthermia for re-treatment of superficial tumour
de Bruijne, 2010 [27]	Evaluation of CEM43CT90 thermal dose in superficial hyperthermia: A retrospective analysis
Oldenborg, 2010 [65]	Elective re-irradiation and hyperthermia following resection of persistent locoregional recurrent breast cancer: A retrospective study
Linthorst, 2012 [66]	The tolerance of reirradiation and hyperthermia in breast cancer patients with reconstructions
Varma, 2012 [67]	Simultaneous radiotherapy and superficial hyperthermia for high-risk breast carcinoma: A randomised comparison of treatment sequelae in heated versus non-heated sectors of the chest wall hyperthermia
Linthorst, 2013 [68]	Re-irradiation and hyperthermia after surgery for recurrent breast cancer
Oldenborg, 2015 [69]	Reirradiation and hyperthermia for irresectable locoregional recurrent breast cancer in previously irradiated area: Size matters
Yahara, 2015 [70]	Definitive radiotherapy plus regional hyperthermia for high-risk and very high-risk prostate carcinoma: Thermal parameters correlated with biochemical relapse-free survival
Ohguri, 2018 [71]	Relationships between thermal dose parameters and the efficacy of definitive chemoradiotherapy plus regional hyperthermia in the treatment of locally advanced cervical cancer: data from a multicentre randomised clinical trial
Kroesen, 2019 [72]	The effect of the time interval between radiation and hyperthermia on clinical outcome in 400 locally advanced cervical carcinoma patients
Datta, 2021 [13]	Quantification of thermal dose in moderate clinical hyperthermia with radiotherapy: a relook using temperature–time area under the curve (AUC)
Nakahara, 2022 [73]	Intensity-modulated radiotherapy with regional hyperthermia for high-risk localized prostate carcinoma
Schem, 2022 [74]	Long-term outcome in a phase ii study of regional hyperthermia added to preoperative radiochemotherapy in locally advanced and recurrent rectal adenocarcinomas

**Table 2:** Summary of the investigated characteristics.

Category	Count (*n*)	Percentage (%)	Mean, Standard Deviation
Hyperthermia and radiotherapy treatment characteristics			
Number of patients	4107		85.6 ± 94.1 *
Radiotherapy dose (Gy)			43.2 ± 13.8
Chemotherapy			
→ no	43	90	
→ yes	5	10	
HT treatment			
→ superficial	33	69	
→ deep	14	29	
→ superficial and deep	1	2	
HT system			
→ microwave	23	48	
→ radiofrequency	8	17	
→ capacitive	4	8	
→ ultrasound	3	6	
→ combination	10	21	
HT duration (min)			56.5 ± 16.0 *
→ superficial HT			53.7 ± 11.7
→ deep HT			63.0 ± 22.9
Number of HT sessions per week			
→ 1 and/or 2	43	90	
→ 3	2	4	
→ not reported	3	6	
Number of HT sessions per patient			5.4 ± 1.9 *
Sequence of treatments			
→ HT before RT/CT	7	15	
→ HT after RT/CT	33	69	
→ other	3	6	
→ not reported	5	10	
Time interval between RT and HT treatment (min)			
→ 0–30	18	38	
→ >30–60	18	38	
→ >60	1	2	
→ not reported	11	22	
Temperature acquisition characteristics			
Thermometry			
→ invasive	48	100	
→ superficial	30	64	
Thermometry technique			
→ thermocouple	11	23	
→ thermistor	7	15	
→ fibreoptic	9	18	
→ combination	14	29	
→ not reported	7	15	
Invasive or minimally invasive thermometry placement			
→ intratumour	25	52	
→ intraluminal	9	19	
→ interstitial	9	19	
→ various	2	4	
→ not reported	3	6	
Temperature acquisition			
→ not continuous	3	6	
→ mapping	10	21	
→ continuous	11	23	
→ combination	11	23	
→ not reported	13	27	
Temperature acquisition rate (min)			
→ 0–1	11	23	
→ >1–5	11	23	
→ >5	6	12	
→ not reported	20	42	
Number of invasive probes			2.3 ± 1.0 *
Number of invasive sensors			9.5 ± 7.2 *
Number of superficial probes			Not reported
Number of superficial sensors			10.9 ± 12.3 *
Association of temperature descriptors with treatment outcome and toxicity			
Treatment outcome			
→ no association	13	27	
→ association	30	63	
→ not reported	5	10	
Toxicity			
→ no association	17	35	
→ association	7	15	
→ not reported	24	50	

* Mean for all 48 studies.

**Table 3:** Template to report HT system and thermometry characteristics, as well as temperature and thermal parameters.

Category	Description		
HT system (and applicator)	e.g., 8 MHz radiofrequency capacitive system [62]		
Coupling method	e.g., water or mineral oil [33]; 5% NaCL [62]		
Temperature of cooling liquid	e.g., mean of 39 °C		
	Invasive	Superficial	
Thermometry system (uncertainty)	e.g., thermistor (±0.2 °C)	e.g., thermistor (±0.2 °C)	
Invasive thermometry placement	e.g., intraluminal	Not applicable	
Temperature acquisition	e.g., continuous and stationary in the bladder and rectum e.g., mapping with a step size of 1 cm and mean map length of 14 cm	e.g., skin surface of the buttocks and abdomen	
Temperature acquisition rate (min)	e.g., every 5 min or continuous	e.g., every 5 min or continuous	
Number of probes and sensors per probe	e.g., 2 probes in the rectum and bladder (each probe with 3–5 sensors)	e.g., 2 probes on the skin surface of the buttocks and 1 probe in the abdomen (each probe with 3–5 sensors)	
Total number of sensors	e.g., mean of 8 sensors e.g., mean of 14 sensors within mapping	e.g., mean of 8 sensors e.g., mean of 14 sensors within mapping	
Sampling rate	number of sensors per area/volume of target	e.g., number of sensors per area of target	
	Invasive	Superficial	Total
**Temperature and thermal dose parameters**	T90 Tmin Tmean Tmax CEM43 TRISE AUC	T90 Tmin Tmean Tmax	T90 Tmin Tmean Tmax CEM43

## Data Availability

The data presented in this study are available in the Supplementary Materials.

## Supplementary Materials

The following Supporting Information can be downloaded at: https://www.mdpi.com/article/10.3390/cancers14194795/s1, Table S1: Databases searched for research articles; Table S2: Risk of bias assessment using the QUIPS tool; Table S3: Characteristics from studies included in the review.
